# Has the Australian Endemic Grey Falcon the Most Extreme Dietary Specialization among all *Falco* Species?

**DOI:** 10.3390/ani12121582

**Published:** 2022-06-19

**Authors:** Jonny Schoenjahn, Chris R. Pavey, Gimme H. Walter

**Affiliations:** 1 School of Biological Sciences, The University of Queensland, Brisbane, QLD 4072, Australia; g.walter@uq.edu.au; 2 CSIRO Land and Water, Winnellie, NT 0822, Australia; chris.pavey@csiro.au

**Keywords:** bird of prey, diet, *Falco hypoleucos*, food specialist, raptor, threatened species

## Abstract

**Simple Summary:**

The diet of an animal is one of the most informative aspects of how it interacts with its environment. A clear understanding of a species’ diet is, therefore, crucial for conservation considerations. The Grey Falcon is a rare and threatened raptor, found only in Australia’s vast arid and semi-arid zone. Its diet is subject to dispute, therefore, we studied, through direct observation during more than 17 years of fieldwork, the food that these birds ingested. We found that Grey Falcons of all ages fed almost exclusively on birds. No other food type was ever taken with any regularity. Our results suggest strongly that the Grey Falcon, throughout the year, throughout its life, and across its vast distribution, feeds almost exclusively on birds. We compared our results with the diets of the other species in the genus (*Falco*) and found that the Grey Falcon’s diet is the most extreme, more so than the diet of even those falcon species that are commonly considered to take exclusively birds, such as the Peregrine Falcon. Our evolutionary explanation of the unique dietary specialization of the Grey Falcon takes into account aspects of the species’ environment and relative prey availability.

**Abstract:**

A clear understanding of a species’ diet is crucial in understanding its spatio-temporal dynamics, and is, therefore, pertinent to conservation considerations. The diet of the Grey Falcon (*Falco hypoleucos*), a rare and threatened predator endemic to the Australian arid and semi-arid zone, is subject to diverging assertions; therefore, we studied its diet through direct observation of food ingestion during more than 17 years of fieldwork across the species’ distribution. We found that Grey Falcons of all ages fed almost exclusively on a single type of food, namely, birds, and non-avian food items never constituted a substantial portion of any individual’s diet. The extraordinary circumstances that were associated with the ingestion of non-avian food suggest strongly that, across its vast distribution, throughout the year, and throughout its life, the Grey Falcon feeds almost exclusively on birds. Further, we compared the diets of all *Falco* species and found that the dietary specialization is most extreme in the Grey Falcon, more so than even in the Taita (*F. fasciinucha*) and Peregrine Falcons (*F. peregrinus*). Based on aspects of the species’ environment and relative prey availability, we offer an evolutionary explanation of the apparently unique dietary specialization of the arid-adapted Grey Falcon.

## 1. Introduction

The diet of an animal is one of the most important aspects of its ecology. The knowledge of an animal’s diet informs the study of its physiology, morphology, behaviour, movements, and distribution. Its diet is also pertinent to conservation considerations because environmental changes may affect the distribution and composition of the food available at any particular time and place, and may, therefore, affect the animal’s physical condition, reproduction, and spatio-temporal dynamics. Further, the diet and distribution of top predators, including raptors of the orders Accipitriformes, Falconiformes, and Strigiformes, continue to attract considerable attention because their conservation depends on ensuring that the appropriate prey items are available to them. Sites where these predators are found tend to exhibit high biodiversity, and are, therefore, of particular conservation interest [1].

The species of the genus *Falco* (Falconiformes) are almost exclusively predatory, and all include birds in their diet to some extent [2]. The degree to which birds are incorporated in the diet varies considerably within and between species, and may vary seasonally, locally, and opportunistically. For example, Peregrine Falcons (*F. peregrinus*) specialise on birds [3], Eleonora’s Falcons (*F. eleonorae*) take mainly insects but exploit, during their breeding season, birds on their autumn migration [4], and the diet of the Bat Falcon (*F. rufigularis*), consisting mainly of bats, birds, and insects, may vary greatly with relative prey availability [5].

The general view of the Grey Falcon (*F. hypoleucos*) diet is that this species feeds on birds, small mammals, lizards, and large insects [2,6]. No comprehensive study has, however, been devoted to the feeding of these birds, and the general statements made about their diet seem to suggest that each of those four groups of animals is an important food source for the Grey Falcon. A review of what direct evidence is available in the literature, which comprises predominantly anecdotal reports, revealed that the Grey Falcon feeds virtually exclusively on birds [7]. To elucidate this seeming discrepancy we studied, through direct observation during more than 17 years of fieldwork across the species’ distribution, the food of Grey Falcons of all ages.

## 2. Materials and Methods

This study is part of an ongoing research project that commenced in 2004. The particulars in this section are taken from previous publications that pertain to the same research project [7,8,9,10].

### 2.1. Study Species

The Grey Falcon is a rare Australian endemic raptor. It is a medium-sized falcon with a median body mass of adults captured from the wild of 412 g (N = 7; range 339–448 g) for males and 502 g (N = 9; range 486–582 g) for females [11]. The species is listed nationally and internationally as threatened, category Vulnerable, based on an estimated population size of fewer than 1000 mature individuals [12,13,14]. The species is distributed at very low density across parts of Australia’s vast arid and semi-arid zone, an area of about 5 million km^2^ or 70% of the Australian mainland [15,16,17]. Specifically, the species is restricted to areas that are classified as ‘hot desert’ and ‘hot savannah’, the hottest climate zones recognised by the Köppen–Geiger Climate Classification [17,18]. The Grey Falcon appears to be the only *Falco* species that is virtually entirely (i.e., all individuals) and permanently confined to arid-hot conditions [7].

### 2.2. Study Area and Study Period

This study was conducted across the arid and semi-arid zones (and adjacent areas) of Australia, thus encompassing the entirety of the species’ distribution [7]. Sites are undisclosed to protect this threatened species. Data were collected between March and November of the years 2004 to 2021, starting on 27 July 2004 and concluding on 20 September 2021.

### 2.3. Data Collection

Total observation time was 2561.0 h, involving a total of 149 year-sites, as follows. A site that was visited multiple times in a given year was counted as one, and a given site visited in N years was counted as N. Observations involved one or more free-flying individuals of the same age-group, breeding pairs with or without hatched young, and adult(s) closely associated with their ≤1-yr-old offspring (for the prolonged juvenile dependence of Grey Falcons on their parents see [10]). All observations were carried out from the ground with binoculars (10 × 42 BN, Leica, Wetzlar, Germany) and telescope (Apo-Televid 77 with 32 × WW eyepiece, Leica, Wetzlar, Germany), and were conducted exclusively by a single observer (J.S.), thus ensuring the consistency of the collected data. To not disturb the falcons, observations were carried out at distances ranging typically from 150 m to 300 m, which at times hampered the identification of the food item. Further, some taxa are easier to identify under those observation conditions than other taxa, such as, for example, Budgerigar (*Melopsittacus undulatus*). In consequence, a break-down of food items to below class level was outside the scope of this article. Reported previously were finches, doves, pigeons, parrots, and cockatoos, among other species, with body masses ranging from 12 g (Australian Zebra Finch (*Taeniopygia castanotis*)) to about 300 g (Galah (*Eolophus roseicapilla*)) [7].

The results on Grey Falcon diet were then compared with the diets of all the other *Falco* species, taken from [2] and focused single-species studies.

## 3. Results

We recorded Grey Falcons ingesting 551 food items, involving 87 year-sites distributed across the species’ distribution (Table 1). We identified 237 food items (43%) to class level, involving up to 257 individual Grey Falcons from across the species’ distribution (Table 1 and Table 2). All except three identified food items were birds (N = 234, 99%), and they were ingested by Grey Falcons of all ages, nestling to adult (Table 2). The remaining items were two small mammals [17], and a lizard [10]. Our observations on non-avian food items are augmented by those of other authors in Table 3. This table also describes situations in which non-avian food items could have easily been captured by Grey Falcons, but were not, as well as the circumstances of these incidents.

We then compared the diets of all *Falco* species (Table 4). Table 4 presents, qualitatively, those food types (birds, invertebrates, mammals, reptiles and amphibians combined, and carrion) that form substantial proportions of the food of the respective species. Details on the diets, including references, are provided in Table 5.

## 4. Discussion

Our results show that the Grey Falcon feeds virtually exclusively on birds (Table 1). Non-avian food never constituted a significant proportion of the diet of a Grey Falcon of any age (Table 2). Specifically, the three non-avian food items that we observed been ingested represented a mere 1.3% of the identified food items (Table 1). Of the prey items, 57% remained unidentified; therefore, the overall proportion of non-avian prey may have been greater or smaller. Nevertheless, the large number of identified food items ingested (237, Table 1) and the large number of falcons involved in consuming these items (up to 257, Table 2), from across the species’ entire distribution, indicate that our results are robust.

The circumstances associated with the three non-avian food items suggest that Grey Falcons may take mammals and reptiles, but only opportunistically, and, even then, only under exceptional circumstances, such as when rodents are at a peak of their population irruption and then become partially diurnal. An item so obtained may not even be ingested. For example, the female partner of an actively breeding pair rejected a lizard that was offered to her by the male although her most recent feed at that time may have been 24 or more hours previously (footnote 6 in Table 3).

Observations on the diet of the Grey Falcon during the summer months, i.e., December, January, and February, are lacking. This is because it is hazardous to travel and work at remote locations in that extreme environment in summer. Nevertheless, our results suggest strongly that the Grey Falcon feeds virtually exclusively on birds throughout the year. Our comparison of the diets of all *Falco* species indicates that, of all the *Falco* species for which data are available, the Grey Falcon has the most extreme dietary specialization (Table 4). This includes the Taita Falcon (*F. fasciinucha*) and the Peregrine Falcon, both of which are widely considered to specialise on avian prey. The Taita Falcon feeds mostly on birds, but is known to hunt, and feed on, insects regularly (No. 38 in Table 5, and references provide there). By contrast, the 28 hunts of Grey Falcon recorded during the present study involved birds exclusively [9]. The Peregrine Falcon is often seen as a bird specialist [3,38]. A significant number of studies demonstrate, however, that Peregrine Falcons may include substantial proportions of a wide variety of non-avian animals in their diet. In addition, scavenging has also been reported for this species (for details see No. 37 in Table 5). Whether the great variety of food types that are important to the cosmopolitan Peregrine Falcon is related to the species living across almost all types of environments, and whether certain subspecies take particular food types more frequently than other subspecies, remain questions worth investigating.

The reason for the Grey Falcon’s extreme dietary specialization is not immediately clear. The following consideration of aspects of the species’ evolution, environment and relative prey availability allow an explanation of how this species came to specialise exclusively on birds.

All *Falco* species feed on birds to some extent (Table 4), so it is the strict focus on this food type that needs to be explained. We showed here that Grey Falcons of all ages feed, throughout the year, virtually exclusively on bird prey, and we have shown previously that their hunting method is almost invariably the fast stoop [9]. These two characteristics may well be adaptive in the Grey Falcon. Indeed, the Grey Falcon lacks the capacity of the hover-and-drop hunt that is used, for example, by the Nankeen Kestrel (*F. cenchroides*) to capture reptiles and small mammals on the ground [36,39,40,41,42]. Similarly, the comparatively short legs of the Grey Falcon [42] appear unsuited to stalking prey on the ground, as does, for example, the Brown Falcon (*F. berigora*) when hunting reptiles [43].

In regard to environment, the Grey Falcon is the most specialised *Falco* species; Grey Falcons breed and live virtually exclusively in the hot arid and semi-arid zone of Australia [7]. We assume that the Grey Falcon speciated in an environment similar to the one it now inhabits [7]. We assume, further, that its ancestral population also hunted birds, like its closest living relatives, which include, for example, the Taita and Peregrine Falcons [44]. In the arid environment inhabited by the Grey Falcon, avian prey species are active throughout the day, which is ecologically significant to this predator. For example, potential prey birds drink at open waterbodies even during the warmest period of the day, and this includes species that are important food sources for the Grey Falcon, such as the Australian Zebra Finch and the Diamond Dove (*Geopelia cuneata*) [7,45]. Indeed, the feeding frequency at Grey Falcon nest sites was higher between 0900 and 1500 local solar time than outside that period, and this was independent of the maximum daytime temperature [9]. At high temperatures in arid Australia all other organisms that are potential alternative prey to a falcon are not so readily and, importantly, not so reliably available. Reptiles mostly seek shade during periods of high heat loads, insect concentrations (which are exploited by, e.g., the Lesser Kestrel (*F. naumanni*)) are less frequent and expansive than they are, for example, in parts of Africa, southern Europe and western Asia, and mammals are mostly nocturnal [46,47,48,49,50].

The ancestral Grey Falcon population may have, thus, become refined, in an environment similar to the one that is now inhabited by the Grey Falcon, to prey predominantly on birds and use the fast stoop, which is, evidently, well-suited to capture birds. For the Grey Falcon, birds are, evidently, readily obtainable throughout the year and provide an absolutely sufficient food source *if* present in adequate numbers. The latter requirement would explain the partly nomadic behaviour of at least some of the individuals of this species [7].

## 5. Conclusions

In summary, preadaptation, in combination with the extreme circumstances of the environment to which the species adapted, moulded an array of morphological and behavioural characteristics in the Australian arid-adapted Grey Falcon, including its extreme, and apparently unique, dietary specialization.

## Figures and Tables

**Table 1:** Number of food items observed being ingested by Grey Falcons (*Falco hypoleucos*), presented separately for each Australian State and Territory involved in this study. The identified items are also sorted by animal group, and the relevant percentages are provided as well as the totals for the whole of Australia.

State or Territory	Number of Year-Sites *	Number of Items Observed Being Ingested	Number of Items Identified to Class Level	Birds	Mammals	Lizards
New South Wales	1	1	1	1 (100%)	0 (0.0%)	0 (0.0%)
Northern Territory	13	109	67	66 (98.5%)	0 (0.0%)	1 (1.5%)
Queensland	44	264	100	98 (98.0%)	2 (2.0%)	0 (0.0%)
South Australia	6	38	14	14 (100%)	0 (0.0%)	0 (0.0%)
Western Australia	23	139	55	55 (100%)	0 (0.0%)	0 (0.0%)
Total	87	551	237	234 (98.7%)	2 (0.8%)	1 (0.4%)

* For the definition of year-site see Methods section.

**Table 2:** Frequencies of food items observed being ingested by Grey Falcons in different age groups (same data as Table 1). The item numbers, and their relevant percentages, are sorted by animal group. Any given food item may have been consumed (partly) by more than one Grey Falcon *, and the largest possible number of Grey Falcon individuals that may have fed on the item was used in the tallies shown below.

Age Group	Number of Food Items Ingested	Number of Grey Falcons Involved in All Feedings Combined	Number of Grey Falcons Involved in Feeding on Identified Items	Number of Ingested Items Identified to Class Level	Birds	Mammals	Lizards
Nestling, fledgling	491	145	119	194	191 (98.5%)	2 (1.0%)	1 (0.5%)
Juvenile, immature, yearling	35	10	7	18	18 (100%)	0 (0.0%)	0 (0.0%)
Adult	550	166	131	237	234 (98.7%)	2 (0.8%)	1 (0.4%)

* One food item was, for example, consumed partially by six individuals: the adult male brought a partly eaten item to the nest, the female took the item and fed it to the three nestlings and a dependent yearling, and also ingested parts of the item herself [10].

**Table 3:** Observations of Grey Falcons and their behaviour towards confirmed non-avian food items (N = 5), and possible such items (N = 3). The latter list includes those observations in which non-avian food items could readily have been taken but were not. All collated from observations reported here and elsewhere in the literature. (Adapted, with modification, from Supplement 2 of [10], available under the Creative Commons CC-BY-NC licence.)

No.	Year	State or Territory	Item	Involvement of Grey Falcon(s)	Hunt Involved	Item Consumed	Observer	Reference
Observations in which non-avian food items were ingested								
1	2011	QLD	Presumed Long-haired Rat	Delivered to nest by adult male	Not observed	Yes ^1^	J.S.	[17]
2	2011	QLD	Presumed Long-haired Rat	Delivered to nest by adult male	Not observed	Yes ^2^	J.S.	[17]
3	2014	SA	Presumed Long-haired Rat	Captured at base of tree on which the adult falcon was perched	Opportunistic	Almost certainly ^3^	E.D. Moore	[19]
4	1971	QLD	Small lizard (≤250 mm)	Captured by the adult male of a family of 4 (2 adults, 2 young)	Opportunistic	Yes ^4^	G. Czechura	[20]
5	2016	NT	Small lizard (≤250 mm)	Adult male caught lizard dropped by a kestrel	No	Yes ^5^	J.S.	[10]
Observations in which non-avian food items could readily have been taken but were not								
6	2020	WA	Small lizard (~250 mm)	Delivered to nest by adult male	Not observed	No ^6^	J.S.	[10]
7	2010	QLD	Locust swarms	Adult male and female ignored the locust swarms	No	No ^7^	J.S.	[11]
8	2021	WA	Grasshopper	The grasshopper collided with a low-flying juvenile falcon	No	Probably not ^8^	J.S.	[10]

Abbreviations: ‘NT’, Northern Territory; ‘QLD’, Queensland; ‘SA’, South Australia; ‘WA’, Western Australia. ^1^ A rat-sized mammal was delivered to the nest by the breeding adult male at 28 min before sunset. The item was likely a Long-haired Rat (*Rattus villosissimus*) as that species was extremely abundant in the area at the time, and active at night as well as during the day (which is unusual and occurs particularly when the rodents are extremely abundant). The item was subsequently fed to the nestlings by the adult female. Note: the body mass of Long-haired Rat ranges from 60 to 280 g, average 134 g [21]; this is inside the range of body mass of the bird species that have been recorded as food of the Grey Falcon. ^2^ As above, but involving a different pair and nest site, and the time was 1 h 20 min before sunset. ^3^ “The Grey Falcons perched out of sight from the observer. After a few minutes one dropped to the ground at the base of a Coolibah [Tree] ~10 m from where it had perched, pounced onto a small rat-like mammal and captured it, then flew with the prey in its talons…” [19] (p. 30). We presume that the item was subsequently consumed by the observed Grey Falcon or a closely associated family member. ^4^ “… a lizard (either a dragon, *Lophognathus* or pygmy monitor *Varanus*) … was captured after one of the four birds (adult pair and two young) left its perch and descended to the ground in a slow, shallow dive.” [20] (p. 10). “My impression was that the lizard was an opportunistic prey item. Date was between late December 1971 and early January 1972 and immediately after the passing of a Tropical Cyclone [TC Althea] through that area.” (G. Czechura pers. comm. to J.S., 16 February 2011). The behaviour of these Grey Falcons was likely influenced by the presumed temporary shortage or disturbance of avian prey in the aftermath of the cyclone. Note that size of the lizard was stated by [20] (p. 10) as “large”, by [22] (p. 12) as “large, (c. 0.5–0.75 m)”, and by G. Czechura in pers. comm. to J.S. on 16 February 2011 as “c. 200–250 mm (total length) and slender”. We consider the latter description to be the most accurate. Note: body mass of a lizard of that description, e.g., *Lophognathus horneri*, is <30 g (databank of the Western Australian Museum, R. Johnstone pers. comm. to J.S., 26 April 2022); this is inside the range of body mass of the bird species that have been recorded as food of the Grey Falcon. ^5^ The incident took place at the site of an active Grey Falcon nest with three recently fledged young. The nest was about 60 m above ground on a telecommunication repeater tower (‘repeater’), on a metal grid that constituted one of several landings of the repeater’s service ladder. The active nest of a Nankeen Kestrel (*Falco cenchroides*) was on the same landing and about 1.5 m away from the Grey Falcon nest. At midday on the day of the incident the adult male kestrel flew from a westerly direction toward the repeater, carrying a small dead lizard in its talons. At that time the adult male Grey Falcon was perched half-way between the two nests. When the kestrel was about 10 m from the repeater, the Grey Falcon male left his perch into a northerly direction, apparently not as a reaction to the kestrel’s approach. The male kestrel appeared to be startled by the larger Grey Falcon taking flight at the precise moment of his approach, and let go of the lizard. The Grey Falcon took a short quick dive, grasped the lizard in mid-air, returned to the repeater and presented the lizard to one of his offspring, which took the lizard. For about 20 min, the fledgling held the lizard alternatively in its beak and talons, seemingly unsure what to do with it. Eventually the fledgling commenced feeding laboriously and clumsily on the lizard. ^6^ At the site of an active nest, the adult male arrived at 1649 h local time with an unidentified lizard of about 250 mm total length. The female flew from the nest and perched next to the male, which held the lizard in its beak. She uttered begging calls, flew off briefly several times and returned, and did not take the lizard that the male dangled frequently in front of her. This continued for about five minutes. Then the female seized the lizard and let go of it, seemingly deliberately, with a resolute downward movement of her head. The lizard dropped to the ground, which was covered with tall grass, and was apparently never retrieved by either adult Grey Falcon. The delivery of the lizard was the only observed delivery of potential food by the male on that day, with observations conducted from 0922 h (2.5 h after sunrise) to after sunset, which occurred at 1755 h. Provided that we had noticed every feeding event, it appears safe to assume that her most recent feed had occurred on the previous day, because these birds are known to hunt predominantly during the warmer parts of the day and only exceptionally during the first few hours after sunrise [9]. On the next day, at 0710 h (25 min after sunrise), the female captured a Little Button-quail (*Turnix velox*) on the ground about 50 m from the nest, and fed on it. ^7^ “A pair of Grey Falcons was observed flying through and perching amidst substantial swarms of Australian Plague Locust (*Chortoicetes terminifera*) without feeding on these insects.” [11] (p. 76). The pair was not breeding at the time. ^8^ On 22 April 2021 an individual aged about six months after fledging was observed for 45 min at a telecommunication repeater tower in the Pilbara region of Western Australia. The juvenile appeared emaciated with an empty crop, and had below adult-standard motor skills, specifically those involving avoiding obstacles in its flight-path and choosing an appropriate perch. Repeatedly, it stooped clumsily and without success at passerines that moved among bushes along the perimeter fence of the repeater, returning to the repeater between the attacks. After one such unsuccessful stoop it came within half a metre of the ground and hit, seemingly by accident, a grasshopper that had launched itself into the air, presumably in response to the approaching falcon. The grasshopper fell to the ground, the young falcon landed next to it, picked it up with its bill and manipulated it. Whether the young falcon fed on the insect remains unclear.

**Table 4:** The 38 *Falco* species and their diets, presented qualitatively. Indicated are the food types (by taxon) that form important proportions of the diet of each *Falco* species, either generally, temporarily (e.g., seasonally), locally, or otherwise. The specific details upon which each entry is based are provided in Table 5. For *F. fasciinucha* Taita Falcon (No. 38), the food type ‘Invertebrates’ is shaded light-grey to indicate that this species regularly hunts, and consumes, invertebrates, and that the importance of that food type to this species is, as yet, not known. The Taita Falcon is rare and little-studied and its diet is known largely from anecdotal reports (examples are provided in Table 5). *Falco hypoleucos*, the Grey Falcon (No. 30), is highlighted in bold font and black shading. Species names according to [23].

No.	*Falco* Species	Common Name	Birds		Invertebrates		Mammals		Reptiles and Amphibians		Carrion
											
1	*naumanni*	Lesser Kestrel									
											
2	*tinnunculus*	Common Kestrel									
											
3	*newtoni*	Madagascar Kestrel									
											
4	*punctatus*	Mauritius Kestrel									
											
5	*araeus*	Seychelles Kestrel									
											
6	*moluccensis*	Spotted Kestrel									
											
7	*cenchroides*	Nankeen Kestrel									
											
8	*sparverius*	American Kestrel									
											
9	*rupicoloides*	Greater Kestrel									
											
10	*alopex*	Fox Kestrel									
											
11	*ardosiaceus*	Grey Kestrel									
											
12	*dickinsoni*	Dickinson’s Kestrel									
											
13	*zoniventris*	Banded Kestrel									
											
14	*chicquera*	Red-headed Falcon									
											
15	*ruficollis*	Red-necked Falcon									
											
16	*vespertinus*	Red-footed Falcon									
											
17	*amurensis*	Amur Falcon									
											
18	*eleonorae*	Eleonora’s Falcon									
											
19	*concolor*	Sooty Falcon									
											
20	*columbarius*	Merlin									
											
21	*rufigularis*	Bat Falcon									
											
22	*deiroleucus*	Orange-breasted Falcon									
											
23	*femoralis*	Aplomado Falcon									
											
24	*subbuteo*	Eurasian Hobby									
											
25	*cuvierii*	African Hobby									
											
26	*severus*	Oriental Hobby									
											
27	*longipennis*	Australian Hobby									
											
28	*novaeseelandiae*	New Zealand Falcon									
											
29	*berigora*	Brown Falcon									
											
**30**	***hypoleucos***	**Grey Falcon**									
											
31	*subniger*	Black Falcon									
											
32	*biarmicus*	Lanner Falcon									
											
33	*jugger*	Laggar Falcon									
											
34	*cherrug*	Saker Falcon									
											
35	*rusticolus*	Gyrfalcon									
											
36	*mexicanus*	Prairie Falcon									
											
37	*peregrinus*	Peregrine Falcon									
											
38	*fasciinucha*	Taita Falcon									
											

**Table 5:** Details of the diets of the 38 *Falco* species, as taken from [2], and supplemented with details from specific studies as indicated. The diet of the Grey Falcon is derived from this study. Particular attention has been given to *Falco* species that take no more than two food types, and the Peregrine Falcon. *Falco hypoleucos*, the Grey Falcon (No. 30), is highlighted in bold font.

No.	*Falco* Species	Diet
1	*naumanni*	Invertebrates; vertebrates (lizards, rodents and birds) are less important. Ref. [24] reported, from Sicily in Italy, vertebrates at a total of 72.8% in biomass: 22.6% rodents, 36.0% reptiles, 14.2% birds.
2	*tinnunculus*	Mammals, with birds (fledglings) often seasonally important. In Mediterranean and Africa, lizards and insects may predominate or be very important, even in terms of biomass.
3	*newtoni*	Insects, also takes mammals, reptiles, some birds and frogs.
4	*punctatus*	Lizards, augmented with birds, insects.
5	*araeus*	Lizards, and insects, birds, mice.
6	*moluccensis*	Mammals, lizards, insects, birds.
7	*cenchroides*	Invertebrates, also mammals, birds, reptiles.
8	*sparverius*	Insects (c. 60%) and vertebrates; in N Hemisphere deserts, birds may represent 35% (biomass), mammals 32% and lizards 28%.
9	*rupicoloides*	Arthropods, birds, lizards.
10	*alopex*	Insects, mammals, lizards, birds.
11	*ardosiaceus*	Insects and reptiles. Also birds, rodents, bats, frogs, earthworms, many insects, also crabs.
12	*dickinsoni*	Birds, reptiles, insects, rodents, frogs, solifugids, crabs.
13	*zoniventris*	Lizards, augmented by birds and insects.
14	*chicquera*	Birds, bats. Also some rodents, reptiles, insects.
15	*ruficollis*	As for *F. chicquera*, of which *F. ruficollis* was formerly considered a subspecies.
16	*vespertinus*	Insects, wide variety of other invertebrates; chicks may be fed mainly with vertebrates (incl. birds, mammals, reptiles, amphibians)
17	*amurensis*	Insects, also birds and some amphibians. Ref. [25] reported, from Mongolia, also mammals.
18	*eleonorae*	Insects, taken mainly outside migration peak and in winter quarters; birds, especially those on autumn migration. Ref. [26] reported, from Crete in Greece, significant proportions of birds and insects during the breeding season; Ref. [27] reported, from Algeria, exceptionally bats, a gastropod and a fish; Ref. [4] reported, from the Aegean archipelago in Greece, also reptiles and bats.
19	*concolor*	While breeding, mainly birds on migration, occasionally bats and other vertebrates. In winter, insects.
20	*columbarius*	Chiefly birds during breeding season; birds, bats, insects at other times, also rodents.
21	*rufigularis*	Bats, birds, insects. Percentage of bats to birds varies greatly between areas.
22	*deiroleucus*	Birds, bats also important. Ref. [28] reported, from 2 sites each in Guatemala and Belize, that, of 105 prey items brought to nests, 90 (85.7%) were birds, and 15 (14.3%) bats.
23	*femoralis*	Birds as staple diet, with some rodents, bats, insects and lizards; insects numerically important.
24	*subbuteo*	Insects, also many birds; bats and lizards locally important.
25	*cuvierii*	Outside breeding season, mainly insects; when breeding, mainly birds.
26	*severus*	Insects, birds, bats.
27	*longipennis*	Birds, bats, insects.
28	*novaeseelandiae*	Birds, sometimes mammals, insects and lizards, rarely carrion.
29	*berigora*	Mammals, birds, reptiles, amphibians, arthropods, carrion, rarely fish. Some seasonal variation.
30	*hypoleucos*	This study: birds made up 99% (N = 234) of the 237 prey items that could be identified to at least class level. The remaining three items were taken under exceptional circumstances. The 28 hunts recorded during the present study all involved birds.
31	*subniger*	Mammals, birds, insects, carrion; rarely reptiles.
32	*biarmicus*	Birds, augmented by rodents, bats, lizards, insects, and, in deserts, spiders and scorpions.
33	*jugger*	Birds, also wide variety of mammals, reptiles, insects.
34	*cherrug*	Mammals, birds generally less important, lizards locally important; beetles also reported.
35	*rusticolus*	Birds, mammals. Ref. [29] reported, from Greenland, considerable variation in percentage of birds to mammals, with > 75% ptarmigan in 2015 and > 50% squirrel in 2014.
36	*mexicanus*	Mammals, birds. Also reptiles, insects.
37	*peregrinus*	Birds, occasionally mammals (including bats, rabbits and voles), also insects, reptiles, exceptionally fish, two reports of carrion feeding. Ref. [30] reported, from an eyrie in Fiji, predominantly flying-foxes as prey; Ref. [31] reported, from Rankin Inlet in Canada, that Lemming and Arctic Ground Squirrel comprised 33% in prey biomass during two non-peak rodent years; Ref. [32] reported, from southern Africa, that 5 (45%) of 11 successful hunts involved bats; Ref. [33] reported, from coastal beaches in Washington USA, scavenging in 28% of 172 feedings, with insignificant seasonal variation.
38	*fasciinucha*	Birds, also takes a few large insects. Ref. [34] observed, during three single-day visits to a site in Malawi, exclusively hunts that involved insects; Ref. [35] reported, from a nest-site in Uganda, that the species hunts large insects; Ref. [36] recorded, from six nesting events in Zimbabwe, 3 (4.7%) hunts involving insects out of 64 hunts in total. Ref. [37] claimed that Taita Falcons take also bats, and this is, to our knowledge, the only published such claim.

## Data Availability

The data presented in this study are openly available from the Mendeley database at [https://doi.org/10.17632/rprms92mjp.1] [51]. Location data are undisclosed to protect this threatened species.
